# Evidence that Fgf10 offers therapeutic opportunities after hyperoxic lung injury in mice

**DOI:** 10.1186/2194-7791-1-S1-A27

**Published:** 2014-09-11

**Authors:** CM Chao, D Al Alam, S Schermuly, H Ehrhardt, KP Zimmer, S Bellusci

**Affiliations:** Justus-Liebig-Universität, Gießen, Germany; Saban Research Institute, Los Angeles, USA; Excellence Cluster Cardio-Pulmonary System, Gießen, Germany

Bronchopulmonary dysplasia (BPD), a chronic lung disease of preterm infants, is characterized by impaired alveolar growth and pathologic vascularization.

## Aims

To investigate the role of *Fgf10* in alveologenesis and during/after hyperoxic lung injury.

## Methods

- 10 weeks old *Fgf10*^+*/-*^ mice (50% *Fgf10* expression compared to WT) in normoxic condition: Lung function and morphometric analysis.
- BPD model:
- *Fgf10*^+^^*/-*^ and *Fgf10*^*+/+*^*mice* were exposed to 85% O_2_ from P0-P8. Morphometric analysis and α-Actin/vWF staining were performed at P3.
- *Rosa26*^*rtTA/+*^*;tet(O)Fgf10* (gain-of-function) mice were exposed to 85% O_2_ from P0-P8. From P9-P45 the pups were exposed to normoxia and fed either with normal food (control) or doxycycline food (experimental) to activate the transgene *Fgf10.* Morphometric analysis was carried out at P45.
- Tolerance study: *Rosa26*^*rtTA/+*^*;tet(O)Fgf10* and WT mice (both 10 weeks old) were exposed to doxycycline for 2 weeks. Then survival rate, histology, Ki67 and TUNEL staining were performed.

## Results

1. *Fgf10*^*+/-*^ mice under normoxic condition have worse lung function and lung structure compared to WT mice.
2. All *Fgf10*^*+/-*^ mice die from hyperoxic injury due to increased lung injury and vascular malformation.
3. Overexpression of *Fgf10* after hyperoxic injury leads to improvement of lung structure compared to control group without overexpression.
4. *Fgf10* overexpression after hyperoxic injury does not increase mortality and side effects (weight loss, mucosal proliferation due to hypercellularity with no impact on apoptosis) are reversible.

## Conclusion

Fgf10 attenuates hyperoxic lung injury, is well tolerated and should be further studied as a potential therapeutic for BPD.

